# Interventional microadhesiolysis: A new nonsurgical release technique for adhesive capsulitis of the shoulder

**DOI:** 10.1186/1471-2474-9-12

**Published:** 2008-01-29

**Authors:** Kang Ahn, Young-Jin Lee, Eun-Ha Kim, Seung-Min Yang, Tae-Kyun Lim, Yong-Soo Kim, Hyung-Joon Jhun

**Affiliations:** 1 Chronic Pain Management Center, Cha Biomedical Center, Kangnam Cha Hospital, Pochon Cha University, Seoul, Republic of Korea; 2 Taewon Diagnostic Radiology Clinic, Seoul, Republic of Korea; 3 Department of Occupational and Environmental Medicine, College of Medicine, Korea University, Ansan, Republic of Korea

## Abstract

**Background:**

A nonsurgical intervention, interventional microadhesiolysis, was developed to release adhesions in joints and soft tissues. This paper introduces the procedure and evaluates the efficacy of the intervention for adhesive capsulitis of the shoulder.

**Methods:**

Ten patients (five men and five women) with primary adhesive capsulitis of the shoulder were treated at a chronic pain management center in Korea. Three specially made needles are used in interventional microadhesiolysis: the Round, Flexed Round, and Ahn's needles. A Round Needle is inserted on the skin over middle of supraspinatus and advanced under the acromion and acromioclavicular joint (subacromial release). A Flexed Round Needle is inserted two-fingers caudal to the inferior border of the scapular spine and advanced over the capsule sliding on the surface of infraspinatus muscle-tendon fascia. The capsule is released while an assistant simultaneously passively abducts the shoulder to full abduction (posteroinferior capsule release). An Ahn's Needle is inserted on the skin over the lesser tubercle and advanced under the coracoid process sliding on the surface of the subscapularis muscle (subcoracoid release).

**Results:**

After the patients underwent interventional microadhesiolysis, the self-rated pain score or severity declined significantly (*p *< .01), the shoulder range of motion increased significantly (*p *< .01), and joint effusion in the affected shoulder decreased or disappeared in nine of ten patients on magnetic resonance imaging compared to their initial status.

**Conclusion:**

Our findings suggest that interventional microadhesiolysis is effective for managing adhesive capsulitis of the shoulder.

## Background

Adhesive capsulitis of the shoulder is characterized by pain in the shoulder and limitation of glenohumeral movement. It tends to occur in patients older than 40 years of age and most commonly in females in their 50s. It has been reported that adhesive capsulitis lasts 2–3 years [1]. Even after this period, however, resolution may not be complete, and many studies have shown mild to moderate restriction of motion [2].

Various interventions have been developed to treat this annoying condition, including oral medication, corticosteroid injection, physical therapy, nerve blocks, manipulation under anesthesia, distension arthrography, and surgical or arthroscopic release [3-8].

Evidence exists that the pathological changes underlying adhesive capsulitis involve synovial inflammation with subsequent reactive capsular fibrosis, which is the ultimate course of adhesive capsulitis irrespective of the underlying cause [9]. Therefore, it is imperative that adhesions that affect the joint be released. Distension arthrography and surgical or arthroscopic release procedures have been used for this. However, the limitations of these procedures should be considered: readhesion can result from bleeding or oozing during or after the procedure; intervention-related risks, such as bleeding, infection, and pain after procedures, can occur [10]. Despite these limitations, however, randomized controlled trials have provided little evidence to support or dispute the efficacy of these interventions [11].

A nonsurgical intervention, *interventional microadhesiolysis*, was developed to release adhesions in joints and soft tissues. The procedure has been used to relieve adhesive capsulitis of the shoulder. The objectives of this study were to introduce this procedure and evaluate the efficacy of the intervention for adhesive capsulitis of the shoulder.

## Methods

### Subjects

Ten patients (five men and five women) with primary adhesive capsulitis of the shoulder were treated at a chronic pain management center in the Republic of Korea between 2005 and 2006. The diagnosis of primary adhesive capsulitis in the subjects was made by an expert in pain management based on the medical history and physical examination, *i*.*e*., pain in the shoulder and global loss of glenohumeral movement without underlying pathologies such as diabetes mellitus, previous upper extremity fracture, surgery with immobilisation, or stroke.

The average age of the subjects was 56.0 ± 7.9 years: 59.0 ± 8.9 years for the males and 53.0 ± 6.3 for the females. The average pain duration in the subjects was 6.2 ± 2.9 months: 5.0 ± 1.4 months in males and 7.4 ± 3.6 months in females. The adhesive capsulitis affected the left shoulder in four patients (two men and two women) and the right shoulder in six (three men and three women). Table 1 summarizes the patient profile. The Institutional Review Board of Kangnam Cha Hospital, Pochon Cha University, approved the study protocol, and all of the subjects gave informed consent.

**Table 1:** Profiles of the patients who underwent interventional microadhesiolysis

					Glenohumeral ROM			Pre-interventional MRI finding		
							
Patient	Age (years)	Sex	Affected shoulder	Pain duration (months)	Abd (deg)	ER (deg)	IR (deg)	Joint effusion*	Thickness of the joint capsule/synovium (mm)	Stage^¶^
1	56	F	Right	6	90	30	15	++	9.1	3
2	49	M	Right	4	75	30	15	+	4.9	2
3	57	F	Left	3	90	30	15	++	5.3	2
4	51	M	Right	6	80	30	15	++	7.0	3
5	44	F	Left	12	80	45	30	+	5.6	3
6	60	M	Right	6	90	30	15	++	7.6	3
7	49	F	Right	6	90	45	15	++	4.8	2
8	59	F	Right	10	90	30	15	++	4.2	2
9	66	M	Left	3	90	30	15	+	5.0	2
10	69	M	Left	6	90	30	15	++	5.0	2

ROM, range of motion; Abd, abduction; ER, external rotation; IR; internal rotation.

* '+' indicates minimal joint effusion; '++' indicates moderate joint effusion.

^¶^The stage of adhesive capsulitis was designated according to Hannafin and Chiaia (2000).

### Needles

**Figure 1:**
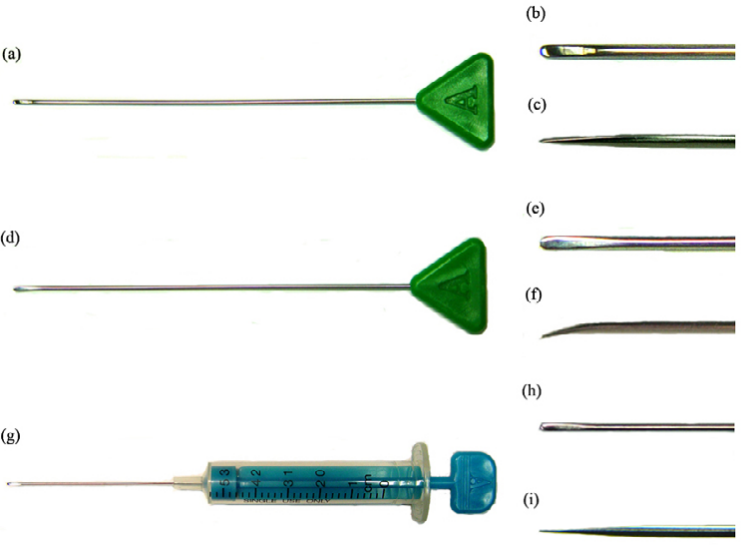
The needles used in interventional microadhesiolysis. (a) The Round Needle and close-ups of its tip from above (b) and the side (c); (d) The Flexed Round Needle and close-ups of its tip from above (e) and the side (f); (g) Ahn's Needle and close-ups of its tip from above (h) and the side (i).

The needles used in interventional microadhesiolysis are shown in Figure 1. They are the Round (Figure 1a), Flexed Round (Figure 1d), and Ahn's (Figure 1g) needles (Hansung Precision, Anyang, Korea). The latter was named after the developer. The Round and Flexed Round needles are 1.2 mm in diameter and 80 mm long. Ahn's Needle is 0.7 mm in diameter and 65 mm long. The Flexed Round Needle is similar to the Round Needle, but its tip is flexed. Ahn's Needle is similar to the Round Needle, but has a syringe-like appearance, with scales on the surface of syringe, and it is thinner and shorter than the Round Needle. Use of these specially made needles is the unique feature of this technique.

In medicine, hollow injection needles are generally used to administer medications into tissues. They are rigid and inflexible, penetrating tissues that are softer than the needle or are stopped if the tissues are harder, which can damage the tissues. Therefore, a new type of needles was developed for interventional microadhesiolysis. They are streamlined, solid, flexible, and have blunt round tips (Figure 1(b),(c),(e),(f),(h),(i)). They were derived from the needle used in intramuscular stimulation (IMS) developed by Gunn [12]. The IMS needle is used to penetrate and release deep contractures. Gunn insists that stimulation by IMS needle lasts for several days and it promotes healing by the release of the platelet derived growth factor [13]. The needles used in interventional microadhesiolysis are thicker than IMS needle (diameter 0.30–0.35 mm) and more rigid, but have flexibility. They have been awarded a patent in Korea (Patent No. 10-2004-41689).

### Procedure

Interventional microadhesiolysis for adhesive capsulitis involves three release approaches: subacromial release, posteroinferior capsule release, and subcoracoid release. The Round Needle is used for the subacromial release, the Flexed Round Needle for the posteroinferior capsule release, and Ahn's needle for the subcoracoid release.

First, the patient is asked to lie on a table in the prone position and is sedated with intravenous propofol (Diprivan^®^, 2–2.5 mg/kg for induction and 100–200 μg/kg/min for maintenance) to avoid pain during the procedure.

**Figure 2:**
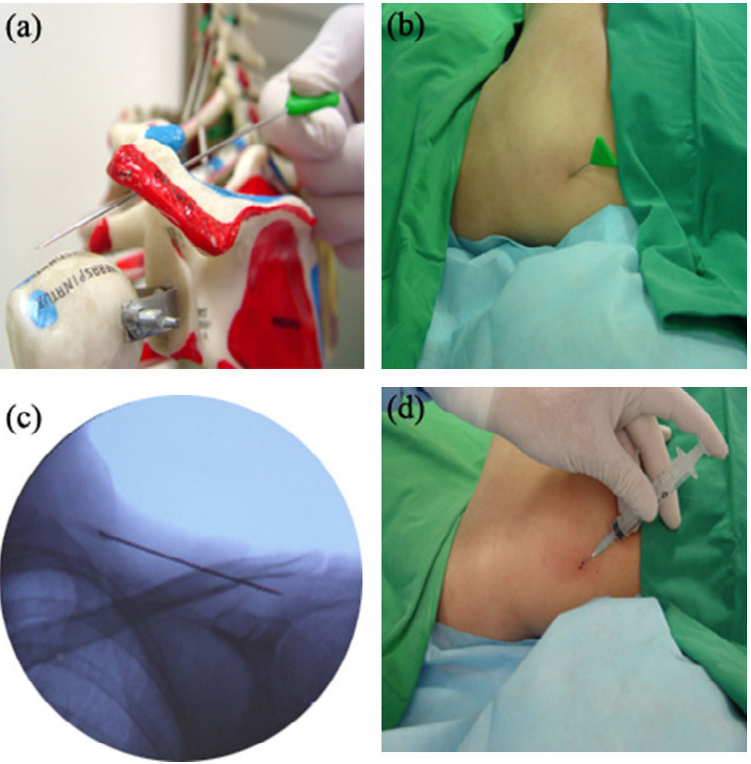
The subacromial release procedure in interventional microadhesiolysis for adhesive capsulitis of the shoulder. (a) A Round Needle inserted at the superior scapular spine of the shoulder beneath the acromion on the surface of the supraspinatus muscle in a human skeleton model; (b) A Round Needle inserted in a patient; (c) The position of the Round Needle inserted in the patient could be identified on fluoroscopy; (d) The injection of diluted triamcinolone acetonide (0.4 mg/ml) after completion of the release to prevent readhesion.

Figure 2 shows the subacromial release procedure. A Round Needle is inserted on the skin over middle of supraspinatus of the affected shoulder and advanced under the acromion and acromioclavicular joint (Figure 2(a),(b)). The needle is moved forward and backward, sliding on the surface of the supraspinatus muscle-tendon fascia along the subacromial space and under the acromioclavicular joint until no resistance is felt at the tip of the needle. When no resistance is felt, this procedure is finished.

**Figure 3:**
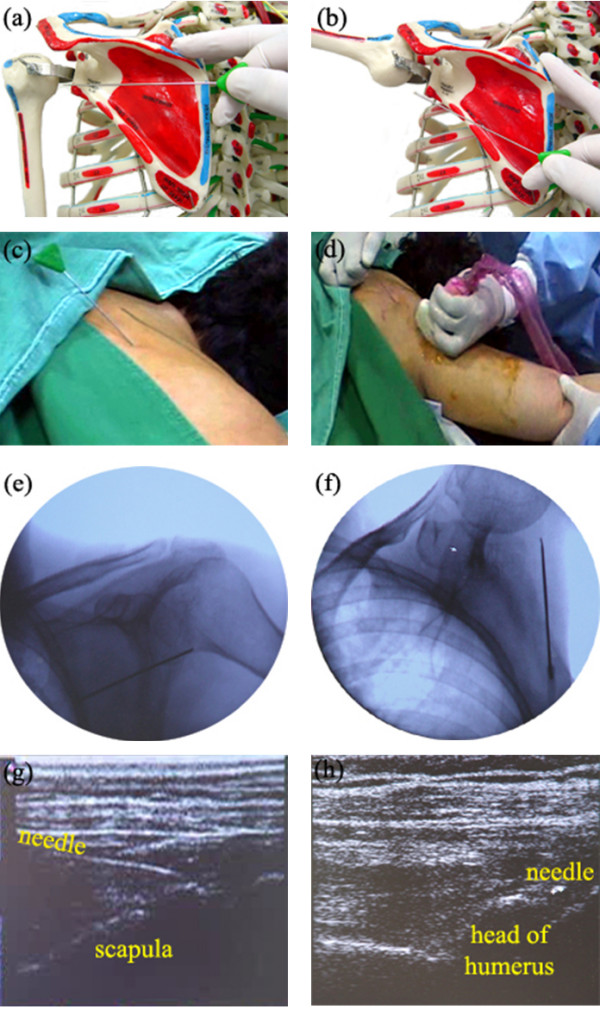
The posteroinferior capsule release procedure in interventional microadhesiolysis for adhesive capsulitis of the shoulder. (a) A Flexed Round Needle inserted below the inferior border of the scapular spine on the infraspinatus muscle-tendon fascia in a human skeleton model; (b) Release of capsular fibrosis using the Flexed Round Needle in a human skeleton model; (c) A Flexed Round Needle inserted in a patient; (d) Release of the capsular fibrosis using the Flexed Round Needle and with simultaneous passive abduction by an assistant; Fluoroscopic images of a Flexed Round Needle inserted in a patient at (e) 45° of abduction and (f) near full abduction; (g) Longitudinal ultrasound image of a Flexed Round Needle inserted in a patient; (h) Transverse ultrasound image of a Flexed Round Needle inserted in a patient.

Figure 3 shows the posteroinferior capsule release procedure. A Flexed Round Needle is inserted two fingers caudal to the inferior border of the scapular spine and advanced over the posterior capsule, sliding on the surface of the infraspinatus muscle-tendon fascia (Figure 3(a),(c)). The needle is moved forward and backward over the posterior capsule until about 90° of abduction is achieved. After 90° of abduction is achieved, the needle is inserted two fingers caudal to the first insertion point, targeting the axillary recess, and also moved forward and backward. While the operator releases the capsular fibrosis using the Flexed Round Needle under ultrasonographic guidance, an assistant gently abducts the shoulder to the point at which no resistance is felt, as obtained with release of the capsular fibrosis. This release and passive abduction process is performed simultaneously, and the degree of abduction is increased gradually (Figure 3(b),(d)). When full abduction of the affected shoulder is achieved, this procedure is finished.

**Figure 4:**
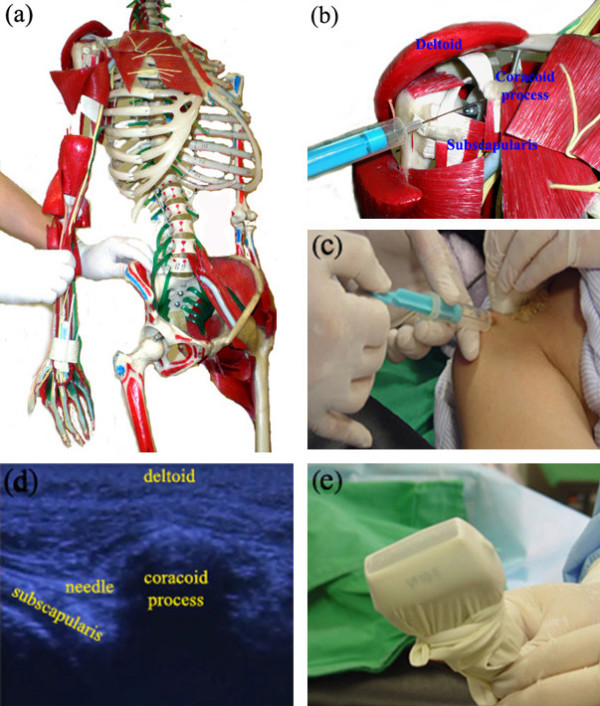
The subcoracoid release procedure in interventional microadhesiolysis for adhesive capsulitis of the shoulder. (a) Positions of the shoulder and elbow during the subcoracoid release procedure, shown using a human skeleton model. The patient's shoulder is adducted, externally rotated, and extended 15–30°, and the elbow is extended; (b) An Ahn's Needle inserted in the skin over the lesser tubercle of the humerus and advanced under the coracoid process, sliding on the surface of the subscapularis muscle in a human skeleton model; (c) An Ahn's Needle inserted in a patient under ultrasonographic guidance; (d) Ultrasonographic finding of the subcoracoid release procedure; (e) A linear ultrasound probe wrapped in a sterile surgical glove to prevent contamination.

Figure 4 shows the subcoracoid release procedure. After the posteroinferior capsule is released, the patient is moved to the supine position. The patient's shoulder is adducted, externally rotated, and extended 15–30°, and the elbow is extended (Figure 4(a)). An Ahn's Needle is inserted through the skin over the lesser tubercle of the humerus and advanced under the coracoid process under ultrasonographic guidance (Figure 4(b),(c)). The needle is moved forward and backward under the coracoid process and on the surface of the subscapularis muscle until no resistance is felt at the tip of the needle. When full internal and external rotation is achieved, all the release procedures have been successfully accomplished.

Fluoroscopy (Figure 2(c), 3(e),(f)) and ultrasonography (Figure 3(g),(h), 4(d)) are used to check the position of the needles and guide them. When using ultrasonography, a linear ultrasound probe is wrapped in a sterile surgical glove to prevent contamination (Figure 4(e)). For proper delivery of ultrasound, ultrasound gel is applied between the linear probe and the surgical glove. Sterile ultrasound gel (Aquasonic^® ^100) is applied between the skin of the patient and the surgical glove. The ultrasound gel applied between the linear probe and the surgical glove does not need to be sterile, but that applied between skin of the patient and the surgical glove should be sterile.

After completing each phase of the release, about 5 ml of diluted triamcinolone acetonide (0.4 mg/ml) is introduced along each microadhesiolysis route to prevent readhesion after the procedure (Figure 2(d)). The solution is made by diluting one vial of triamcinolone acetonide (40 mg) with 100 ml normal saline, which is 1/100 of the usual concentration used in steroid injection therapy.

After the patients undergo the initial intervention, their pain level and range of motion (ROM) are evaluated every 3 weeks. If the patient complains of persistent pain or limited ROM, the intervention is repeated 3 weeks later.

### Outcome measures

We selected three outcome measures to evaluate the treatment effect of interventional microadhesiolysis in adhesive capsulitis: the self-rated pain score or severity, the glenohumeral ROM of the affected shoulder, and magnetic resonance imaging (MRI) of the affected shoulder. These measures were assessed before and after the intervention.

The patients were asked to rate their overall pain level using a visual analogue scale (VAS) consisting of a 10-cm line anchored by two extremes of pain. They were also asked to rate the severity of pain at night, during rest, and on activity to evaluate the pain level in specific situations. The severity was described as 1 = none, 2 = mild, 3 = moderate, 4 = severe, or 5 = very severe pain.

Glenohumeral ROM was measured with the patients in a supine position. To measure glenohumeral internal rotation, the humerus was kept in maximal allowable abduction with the elbow flexed 90° while the examiner's hand prevented the scapula from elevating or tilting anteriorly. The forearm was taken toward the supporting surface of the examining table with the palm facing toward the examining table, moving the extremity into internal rotation. Once a firm end point was felt, the ROM was measured. External rotation ROM was taken from the same starting position as for the internal rotation ROM measurement. In this measurement, the forearm was taken posteriorly so that the extensor surface of the forearm was moved toward the head of the examining table [14]. The final self-rated pain score or severity and ROM were evaluated 3 months after the initial intervention.

The patients were also examined with a 1.0-T MRI unit (Magnum 1.0T; Medinus, Seoul, Korea). Axial, oblique sagittal, oblique coronal T2-weighted (TR 3500 ms, TE 96 ms), and proton density-oblique coronal or proton density-axial images were evaluated. We did not perform MR arthrography using intra-articular contrast injection because the patients did not want to undergo an invasive diagnostic procedure before they underwent the invasive treatment, i.e., interventional microadhesiolysis. The radiological diagnosis was based on reported MR findings in adhesive capsulitis [15-17]: thickening of the inferior glenohumeral ligament (IGHL) at the axillary pouch level, the thickness of the joint capsule and synovium, and the amount of joint effusion.

Thickening of the IGHL and a minimal or moderate joint effusion were detected on the MRI obtained before intervention. The thickness of the joint capsule and synovium was measured on the oblique coronal T2-weighted image. The minimum thickness was 4.2 mm and the maximum was 9.1 mm (Table 1). An independent radiologist who had no conflict of interest evaluated the MRI findings in the patients. The follow-up MRI was taken 9.0 ± 3.0 months (range 3–13 months) after the initial intervention.

Wilcoxon's signed-ranks test was used to evaluate the statistical significance of the mean pre- and post-interventional self-rated pain score or severity and the ROM of the affected shoulder. The MRIs of the affected shoulder taken before and after intervention were compared.

## Results

After the patients underwent interventional microadhesiolysis, their self-rated pain score or severity declined significantly compared to their initial status (*p *< .01). Their mean VAS pain score decreased 5.2 ± 2.0 points (range 2–8 points). The severity of pain at night decreased 3.0 ± 1.3 points (range 0–4 points), that at rest decreased 1.2 ± 0.8 points (range 0–2 points), and that on activity decreased 1.8 ± 0.6 points (range 1–3 points). Conversely, the ROM increased significantly (*p *< .01). The angle of abduction increased 85.5 ± 15.0° (range 60–105°), that of external rotation increased 44.5 ± 18.8° (range 15–60°), and that of internal rotation increased 33.0 ± 9.5° (range 15–45°) (Table 2).

**Table 2:** Mean pre- and post-interventional self-rated pain score or severity and range of motion (ROM) of the affected shoulder

Variable	Pre	Post	*p*-value
Self-rated pain score or severity			
Overall^a ^(VAS)	7.6 ± 1.0	2.4 ± 1.8	.0020
Pain^b ^at night	4.2 ± 1.3	1.2 ± 0.6	.0039
Pain^b ^at rest	2.4 ± 1.0	1.2 ± 0.4	.0078
Pain^b ^on activity	3.6 ± 0.5	1.8 ± 0.4	.0020
Glenohumeral range of motion (deg)			
Abduction	86.5 ± 5.8	172.0 ± 11.4	.0020
External rotation	33.0 ± 6.3	77.5 ± 17.2	.0020
Internal rotation	16.5 ± 4.7	49.5 ± 10.1	.0020

^a^Evaluated using the visual analogue scale (VAS) pain score.

^b^Evaluated using the self-rated severity of pain (1 = none, 2 = mild, 3 = moderate, 4 = severe, 5 = very severe pain).

**Figure 5:**
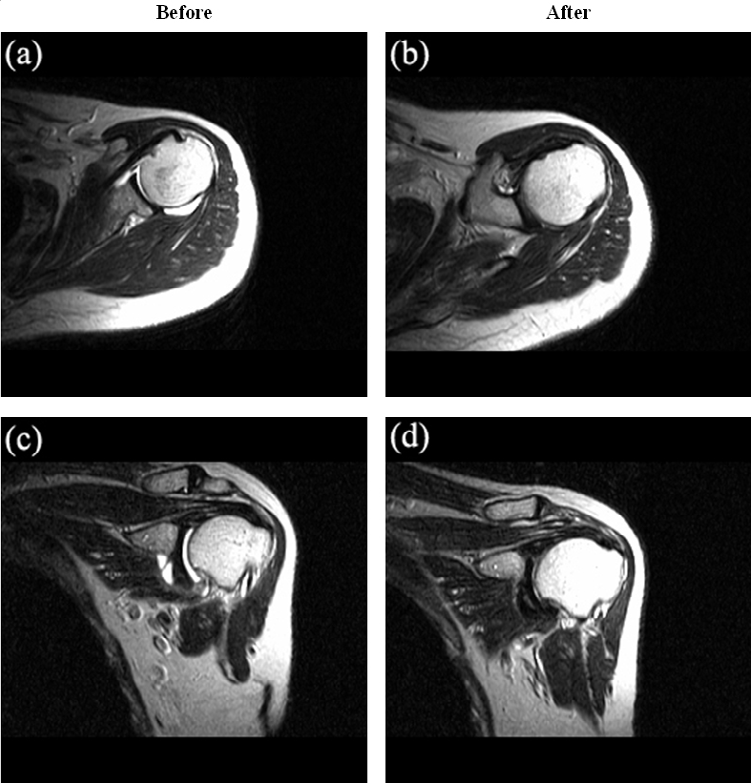
Magnetic resonance images (MRI) of a patient before and after interventional microadhesiolysis. The joint effusion decreased after the patient underwent interventional microadhesiolysis. (a) Axial and (c) coronal views of the shoulder MRI taken before intervention. (b) Axial and (d) coronal views of shoulder MRI taken after intervention.

The joint effusion in the affected shoulder decreased or disappeared in nine of ten patients following interventional microadhesiolysis on the follow-up MRI. It was the most obvious MRI finding after intervention among the patients (Figure 5). However, no significant change was noted in the IGHL thickness. We could not measure the thickness of the joint capsule or synovium on the follow-up MRI because the joint effusion decreased or disappeared.

The patients underwent an average of 1.8 ± 0.6 interventions (range 1–3). Except for the pain after waking following sedation, no significant or fatal adverse effect was noted. Fentanyl 50 μg/h patch (Durogesic^® ^D Trans) was prescribed to the patients who complained of severe pain after waking.

## Discussion

After the patients underwent interventional microadhesiolysis, the self-rated pain score or severity declined significantly (*p *< .01), the ROM of the affected shoulder increased significantly (*p *< .01), and the joint effusion in the affected shoulder decreased or disappeared in nine of ten patients on MRI compared to their initial status. These results suggest that interventional microadhesiolysis is effective for managing adhesive capsulitis of the shoulder.

We used interventional microadhesiolysis clinically after we confirmed its safety and effectiveness in a cadaveric examination. The shoulders of cadavers are stiff irrespective of the presence of adhesive capsulitis before death. We used needles following our described procedure and confirmed that the technique did not cause serious damage and that the shoulders became movable.

Our technique does not include an intra-articular approach. It aims to release the structures that cause significant loss of ROM of the shoulder. It does not aim to release the entire capsule because most studies do not describe significant capsular adhesion as a predominant finding in adhesive capsulitis. Although the glenohumeral joint synovial capsule is often involved in adhesive capsulitis, most of the significant loss of ROM results from pathology in structures outside of the glenohumeral joint synovial capsule (*e.g*., coracohumeral ligament, rotator interval, subscapularis muscle, and subacromial bursa) [18]. The posteroinferior capsule was selected for release because the most significant capsular adhesion is observed in this area in patients with adhesive capsulitis [15-17]. The assistant was required to abduct the shoulder to keep the capsule stretched during the posteroinferior capsule release procedure. Adhesions can be released using a needle when the target tissue is stretched. The supraspinatus muscle and subacromial bursa were released during the subacromial release procedure. The coracohumeral ligament, rotator interval (the triangular portion of the shoulder capsule lying between the supraspinatus and subscapularis tendons), and subscapularis muscle were approached and released during the subcoracoid release procedure. The rotator interval was reached by a needle when the patient's shoulder was adducted, externally rotated, and extended 15–30°.

Follow-up MRI showed that joint effusion decreased or disappeared. The increased shoulder mobility after the intervention might have contributed to this finding. However, no significant change was noted in the thickening of the IGHL. Although the clinical condition of the patients improved, it might take more time to regenerate collagen and the follow-up period for MRI might be insufficient to detect overt changes in the thickness of the IGHL.

Three patients underwent one intervention only, six patients underwent two interventions, and one patient underwent three interventions. In our clinical experience, the treatment outcomes of those who had an underlying partial rotator cuff tear or biceps tendinopathy combined with adhesive capsulitis were not favorable and they needed more than one intervention. We also postulate that the underlying shoulder instability contributed to the unfavorable outcome of the initial treatment.

We recommend that all of the procedures in interventional microadhesiolysis for adhesive capsulitis of the shoulder be performed in sequence. The sequential combination of all three release procedures enables full ROM of the affected shoulder joint in all directions.

Interventional microadhesiolysis has several advantages compared to other interventions for adhesive capsulitis. Although this technique is invasive, it is minimally so. A skilled operator and assistant can perform this technique in 30 min for unilateral shoulder. Pre-interventional clinical laboratory tests and imaging diagnosis can be obtained at outpatient clinics and the patient need not be hospitalized for this treatment. Operators can control the extent of adhesiolysis using the specially made needles for interventional microadhesiolysis. We believe that the characteristics of the specially made needles – streamlined shape, being solid but flexible, and having a round, blunt tip – enable the operators target the lesion accurately, reach a wide area, and allow the minute release of adhesions, while minimizing unwanted tissue damage. As in other invasive interventions, we caution that the contraindications of interventional microadhesiolysis include, for example, pregnancy, bleeding tendency, and generalized weakness.

This study was a case series, which poses a limitation, although we observed favorable treatment results. Thus, further studies or randomized clinical trials are needed to evaluate the efficacy of interventional microadhesiolysis compared to other treatment methods for adhesive capsulitis of the shoulder.

## Conclusion

Interventional microadhesiolysis was developed to release adhesions in joints and soft tissues. The procedure was used to treat 10 patients with primary adhesive capsulitis of the shoulder. Three specially made needles (*i*.*e*., Round, Flexed Round, and Ahn's Needles) and three release approaches (*i*.*e*., subacromial, posteroinferior capsule, and subcoracoid release) were used in interventional microadhesiolysis. After the patients underwent interventional microadhesiolysis, selected outcome measures (*i.e*., self-rated pain score or severity, range of motion, and joint effusion on MRI) were improved compared to their initial status. Thus, interventional microadhesiolysis is effective to manage adhesive capsulitis of the shoulder.

## Competing interests

The author(s) declare that they have no competing interests.

## Authors' contributions

KA developed the interventional microadhesiolysis technique and the three specially made needles used in this study. KA and YJL designed the study. EHK collected the data. SMK and TKL were the assistants during the procedure and they also assisted with data collection and processing. YSK interpreted the magnetic resonance findings of the patients. HJJ analysed the data and wrote the manuscript.

## Pre-publication history

The pre-publication history for this paper can be accessed here:


